# The Antibody Response of Pregnant Cameroonian Women to VAR2CSA ID1-ID2a, a Small Recombinant Protein Containing the CSA-Binding Site

**DOI:** 10.1371/journal.pone.0088173

**Published:** 2014-02-04

**Authors:** Anna Babakhanyan, Rose G. F. Leke, Ali Salanti, Naveen Bobbili, Philomina Gwanmesia, Robert J. I. Leke, Isabella A. Quakyi, John J. Chen, Diane Wallace Taylor

**Affiliations:** 1 Department of Tropical Medicine, Medical Microbiology and Pharmacology, University of Hawai'i at Manoa, John A. Burns School of Medicine, Honolulu, Hawai'i, United States of America; 2 The Biotechnology Center, Faculty of Medicine and Biomedical Research, University of Yaoundé 1, Yaoundé, Cameroon; 3 Centre for Medical Parasitology, Department of International Health, Immunology and Microbiology, University of Copenhagen, Copenhagen, Denmark; 4 School of Public Health, College of Health Sciences, University of Ghana, Legon, Ghana; Institut de Recherche pour le Développement, France

## Abstract

In pregnant women, *Plasmodium falciparum*-infected erythrocytes expressing the VAR2CSA antigen bind to chondroitin sulfate A in the placenta causing placental malaria. The binding site of VAR2CSA is present in the ID1-ID2a region. This study sought to determine if pregnant Cameroonian women naturally acquire antibodies to ID1-ID2a and if antibodies to ID1-ID2a correlate with absence of placental malaria at delivery. Antibody levels to full-length VAR2CSA and ID1-ID2a were measured in plasma samples from 745 pregnant Cameroonian women, 144 Cameroonian men, and 66 US subjects. IgM levels and IgG avidity to ID1-ID2a were also determined. As expected, antibodies to ID1-ID2a were absent in US controls. Although pregnant Cameroonian women developed increasing levels of antibodies to full-length VAR2CSA during pregnancy, no increase in either IgM or IgG to ID1-ID2a was observed. Surprisingly, no differences in antibody levels to ID1-ID2a were detected between Cameroonian men and pregnant women. For example, in rural settings only 8–9% of males had antibodies to full-length VAR2CSA, but 90–96% had antibodies to ID1-ID2a. In addition, no significant difference in the avidity of IgG to ID1-ID2a was found between pregnant women and Cameroonian men, and no correlation between antibody levels at delivery and absence of placental malaria was found. Thus, the response to ID1-ID2a was not pregnancy specific, but predominantly against cross-reactivity epitopes, which may have been induced by other PfEMP1 antigens, malarial antigens, or microbes. Currently, ID1-ID2a is a leading vaccine candidate, since it binds to the CSA with the same affinity as the full-length molecule and elicits binding-inhibitory antibodies in animals. Further studies are needed to determine if the presence of naturally acquired cross-reactive antibodies in women living in malaria endemic countries will alter the response to ID1-ID2a following vaccination with ID1-ID2a.

## Introduction

In pregnant women, *P. falciparum*-infected erythrocytes (IE) express the adhesion ligand VAR2CSA that binds to chondroitin sulfate A (CSA) on syncytiotrophoblasts lining the intervillous space (IVS) of the placenta [1]–[4]. As a result, IE accumulate at the maternal-fetal interface causing placental malaria (PM). Pathology resulting from PM increases the risk of maternal anemia and poor pregnancy outcomes, including low birth weight babies due to prematurity and intrauterine growth restriction [5], [6]. In malaria endemic areas, pregnant women produce antibodies (Ab) to VAR2CSA that inhibit the binding of IE to CSA *in vitro* [7], [8], reduce maternal anemia [9], lower placental parasitemia at delivery [10], [11], increase the length of gestation [12], and improve infant birth weight [12]. Thus, Ab to VAR2CSA play an important role in protecting pregnant women from the severe effects of PM.

VAR2CSA is a 350kDa transmembrane protein with 6 Duffy-Binding-Like (DBL) domains, a cysteine-rich inter-domain region (termed CIDR_PAM_) between DBL2X and DBL3X, and a number of inter-domains [13]–[15]. Data support the feasibility of a VAR2CSA-based vaccine for protecting pregnant women. For a surface antigen, VAR2CSA is surprisingly well-conserved, with limited polymorphism and is the main target of protective immunity against PM [16]–[19]. However, the large size of the molecule makes it difficult to produce a vaccine using the entire molecule; accordingly, extensive efforts are being made to identify the region(s) within the molecule that binds CSA.

Originally it was thought that the CSA binding site was conformationally-created by multiple DBL domains [14], [15]; however, several groups recently showed that it is located in the N-terminal DBL2X- CIDR_PAM_ region [20], [21]. The minimal sequence containing the binding site was recently identified as ID1-DBL2Xb, consisting of ID1, DBL2Xb and 93 amino acids from ID2a [22]. A slightly larger construct, termed ID1-ID2a, consisting of the ID1, DBL2Xb, plus the entire ID2a region, can be expressed in higher yields compared to ID1-DBL2Xb and has entered a clinical development program. The ID1-ID2a is considered to be a strong vaccine candidate, because antisera raised against it in rats inhibit the binding of IE to CSA by nearly 100% [22]. Furthermore, when Ab raised against full length VAR2CSA (FV2) in rats are affinity purified on recombinant ID1-ID2a, the purified Ab effectively inhibit IE binding to CSA by essentially 100% [22]. Thus, ID1-ID2a contains the minimal CSA binding site, induces inhibitory Ab in an animal model, contains major epitopes important in inhibition of binding, and can be produced on a large scale.

The natural acquisition of Ab to ID1-ID2a in pregnant women has not been evaluated. Recently, we demonstrated that Cameroonian women, who were PM negative (PM-) at delivery, had significantly higher Ab levels to FV2 throughout pregnancy, and that women with a high proportion of high avidity Ab to FV2 during the second trimester were at reduced risk of having PM at delivery [23]. Using plasma samples from the same group, we found that women with Ab to more DBL domains and allelic variants were also more likely to be PM- at delivery [24]. Accordingly, plasma from the above women, as well as samples from other cross-sectional studies, were used in the current study to determine if pregnant Cameroonian women naturally produce Ab to ID1-ID2a and if Ab to ID1-ID2a correlate with absence of PM at delivery. If so, then measuring Ab to recombinant ID1-ID2a could be used to determine if pregnant women have sufficient immunity to be protected from PM. Our results indicate that the recombinant ID1-ID2a constructs (3D7 and FCR3 strains) used in this study contain epitopes not exposed in the full-length molecule that are detected by IgG antibodies commonly found in Cameroonians. Accordingly, levels of Ab to these ID1-ID2a constructs cannot be used to determine if women are protected from PM.

## Methods

### IRB approval

The archival coded Cameroonian samples used in the current study were exempt from human subject research by the Committee on Human Studies, University of Hawai'i, Manoa. The original studies were conducted according to the Helsinki Declaration principles and approved by the National Ethics Committee, Cameroon, and the Institutional Review Board at Georgetown University. All women participating in the study gave written informed consent, including the use of their blood samples to measure Ab to malaria. Samples from pregnant women in the USA were obtained from the Hawai'i Biorepository, University of Hawai'i, and were also exempt from human subjects research by the Committee on Human Studies, University of Hawai'i, Manoa.

### Plasma samples

Archival plasma samples from three different sites in Cameroon were used. The sites included Ngali II with an entomological inoculation rates of 0.7 infectious bite/person/night [25], Simbok (1.2–1.9 infectious bites/person/night) [26]; and Yaoundé (0.1 to 1.1 infectious bites/person/month) [27]. Archival coded plasma samples from male and female US subjects (n = 24) and US pregnant women (n = 42) were used as controls.

The first set of archival plasma samples were from a longitudinal study conducted between 2001 and 2005 in the rural village of Ngali II and the city of Yaoundé, Cameroon, in which pregnant women were recruited early in pregnancy and followed throughout their pregnancies [25]. Three plasma samples from each pregnant woman collected during the first, second and third trimesters were used (n = 83 Ngali II village, n = 96 Yaoundé) and samples from males (n = 58 Ngali II, n = 35 Yaoundé) were included for comparison. The second set of archival samples was from a cross-sectional study conducted between 1994 to1997 in Simbok, a peri-urban village [26]. Peripheral plasma samples from all males (n = 51) and females (n = 102) of reproductive age (18–35 years old) were used. The gravidity status was unknown, but the women were not pregnant at the time of sample collection. The third set of plasma samples was from a large cross-sectional study (referred to as “City of Yaoundé cross-sectional cohort”) conducted in Yaoundé from 1996 to 2001 [28], [29] and consisted of 1,944 peripheral blood samples collected at delivery. All samples from placental malaria positive (PM+) women (n = 116) and 348 randomly selected samples from PM− women (*i.e.*, ratio of 1∶3 for PM+ to PM−) were selected from women who had ≥3 pregnancies, ≥20 years of age, and had term or premature deliveries. All PM− women had been exposed to malaria since they had Ab to DBL5 (data not shown).

### Diagnosis of placental malaria

Blood smears of maternal peripheral and IVS blood, and impression smears of placental biopsies, were made. Slides were stained with Diff-Quick Stain Kit (IMEB Inc., San Marcos, CA) and read by two microscopists to determine parasitemia. Placental biopsies were fixed in 10% buffered formalin, embedded, stained with hemotoxylin-eosin and examined for parasites. A woman was considered to have PM if IE were detected in blood smears of IVS blood, impression smears of villous tissue, or histological sections of the placenta.

### Recombinant Protein Expression

Recombinant proteins used in this study included ID1-ID2a, DBL1+2, DBL2, DBL3, DBL5 and FV2 produced in Sf9 insect cells using the Baculovirus vector, and DBL1 (3D7 and 7G8 strains) produced in *Pichia pastoris*, as described previously [24]. Sequence information and detailed protocols have been published previously [22]–[24]. Briefly, full length VAR2CSA [FCR3 strain, GenBank: GU 249598] and ID1-ID2a [3D7 strain, GenBank: JQ247428 and FCR3 strain, GenBank: GU 249598][see Figure S1 for ID1-ID2a sequences and sequence boundaries] were inserted into Baculovirus transfer vector pAcGP67-A (BD Biosciences) with a histidine tag on the C-terminal end. Linearized Bakpak6 Baculovirus DNA was co-transfected with pAcGP67-A plasmids into Sf9 insect cells for generation of recombinant virus particles. Then, 10 ml of second amplification was used to infect High-Five cells in 400 ml of serum-free medium (10486, Invitrogen) at 1 million cells/ml. Secreted recombinant protein was harvested from the supernatant and purified using the AKTA-express purification system (GE Health Care).

### Coupling of recombinant proteins for use in the MultiAnalyte Platform (MAP) assay

The MAP assay was previously optimized for quantification of Ab to multiple malarial antigens [30], including VAR2CSA sequences [24]. To determine the optimal concentration of ID1-ID2a, different amounts of the ID1-ID2a recombinant proteins were coupled to 1 million microspheres (SeroMAP beads, Luminex Corp., Austin, TX), including 1.6, 5, and 10 µg of the 3D7 protein and 1, 5, 10, and 15 µg of the FCR3 protein. ID1-ID2 (3D7) at 1.6 µg/million microspheres and ID1-ID2 (FCR3) at 5 µg/million microspheres were found to be optimal. DBL1 (3D7 and 7G8), DBL1+2 FCR3, DBL2 FCR3, DBL3 (FCR3) and DBL5 (FCR3) were coupled at 1 µg/million microspheres [24]; FV2 was coupled at 3 µg protein/million microspheres based on previous optimization studies [23].

### Optimization of the MAP assay

The MAP assay measures Ab to multiple antigens simultaneously, creating the potential that Ab might compete for the antigens. To assess this possibility, microspheres coupled with ID1-ID2a (3D7) and ID1-ID2a (FCR3) were tested either alone or pooled with FV2 for reactivity with plasma from multigravid Cameroonian women (positive control) and pooled US plasma (negative control). Data showed that Ab levels to both ID1-ID2a constructs were similar when used singly or multiplexed with the other ID1-ID2a construct and FV2 (Figure S2).

### Measuring IgG and IgM using the MAP assay

MAP assay was performed as previously described [23], [24], [30]. Briefly, 50 µl of antigen-coupled microspheres (2,000 microspheres/test) were incubated with 50 µl of a 1∶100 dilution of plasma in PBS-1% BSA (phosphate buffered saline containing 1% bovine serum albumin [BSA]) in pre-wetted wells of filter plates (96 well Multiscreen BV; Millipore, Billerica, MA) for 1 hr at 25°C on a rotating shaker at 500 rpm (Microplate Shaker, Lab-line, Melrose Park, IL). Microspheres were washed twice with PBS-0.05% Tween 20 and once with PBS-1% BSA. Then, 100 µl of secondary Ab (R-phycoerythrin-conjugated, Affini Pure F(ab’)_2_ fragment, Goat anti-human IgG Fc fragment specific from Jackson Immunoresearch, West Grove, PA, Cat # 109-116-170) diluted to 2 µg/ml in PBS-1% BSA was added to each well and incubated as above in the dark for 1 hr. Wells were washed as described above, microspheres were re-suspended in 100 µl PBS-1% BSA, and 85 µl of the microsphere suspension was analyzed using a Liquichip M100 reader (Qiagen, Valencia, CA). The reader was programmed to read a minimum 100 beads per spectral address, DD Gate 7500–15000 and 35 sec timeout. The results were expressed as median fluorescence intensity (MFI). Positive and negative controls were run on each plate that included 3 different pools of plasma from 8 Cameroonian multigravidae with high Ab levels to VAR2CSA and pools of plasma from 40 Americans who never travelled to malaria endemic areas. To measure IgM, R-PE-AffiniPure F(ab’)_2_ fragment anti-human IgM Fc secondary Ab was used (Jackson Immunoresearch, West Grove, PA, Cat # 709-116-073) at 1∶250 dilution in PBS-1% BSA.

### Avidity to ID1-ID2a (3D7 and FCR3) and FV2

Samples collected at delivery (or late in the third trimester, i.e., >27 weeks) were used in the avidity assay for women who were seropositive. The avidity assay was performed as previously described [23]. Briefly, plasma was diluted 1∶300, 1∶1,000 and 1∶3,000 in 1% BSA-PBS and 50 µl of diluted plasma was added to six wells (each dilution in duplicate) containing 50 µl of ID1-ID2a and FV2-coupled microspheres (2,000 microspheres/test) and incubated for 1 hr on a shaker. After incubation, 100 µL of 3 M NH_4_SCN in 1% BSA-PBS was added to half of the wells and 100 µL of 1% BSA-PBS was added to the other half. After 30 minutes of incubation, the wells were washed and incubated with secondary Ab, washed, and analyzed by MicroChip 100 reader as described above. The proportion of Ab that remains bound (i.e., percent high avidity Ab) was determined for each dilution by the following formula: (MFI obtained from wells incubated with salt)/(MFI obtained from corresponding control wells) x100 for each dilution. Then, the average for the 3 dilutions was determined. Positive and negative controls were included on each plate.

### Statistical Analysis

Antibody positivity to ID1-ID2a in Cameroonian males and pregnant women was calculated using a cut-off value equal to the mean +2 standard deviations for US controls (n = 66). The cut-off value for seropositivity to FV2 (FCR3) was calculated for each study site using the mean +2 standard deviations for Cameroonian males residing at each site. Occasional males (n = 2 Yaoundé, n = 1 Ngali, n = 5 Simbok) with MFI greater than the mean +3 SD were excluded from the cut-off calculations. For each continuous variable (such as Ab levels or proportion of high avidity Ab) individual values, their median, lower and upper quartiles, and the interquartile range (IQR) were presented for the study samples as a whole and for relevant subgroups. Two-sample t-tests (or Mann-Whitney tests when data was not normally distributed) were used to compare between Cameroonian males and pregnant women and between PM+ and PM− samples. Paired t-test was used to compare IgM levels to FV2 and ID1-ID2a of the same individuals. For repeated measures data, e.g., IgG levels during pregnancy, the non-parametric Friedman test was implemented to detect differences at different sites during pregnancy. For the comparison of Ab prevalence and prevalence of high avidity Ab between Cameroonian males and females, two-tailed Fisher's exact tests were performed. Finally, to evaluate the association between the reactivities of Ab to ID1-ID2a and to FV2, correlation analysis was utilized and Pearson's correlation coefficients (r) were calculated. Two-tailed p<0.05 was considered statistically significant. Data were analyzed using GraphPad Prism 6.0 and SmallStata 12.

## Results

### IgG levels to ID1-ID2a in Cameroonian women during pregnancy

**Figure 1:**
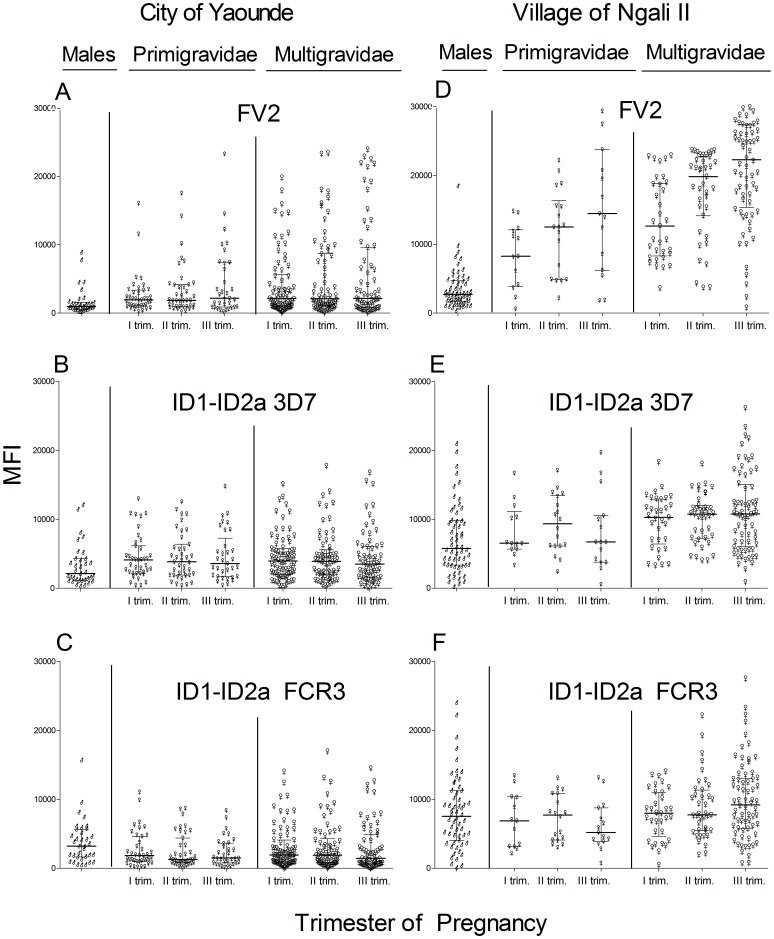
IgG levels to ID1-ID2a in Cameroonian women during pregnancy. Ab levels to FV2 (**A, D**), ID1-ID2a 3D7 (**B, E**), and ID1-ID2a FCR3 (**C, F**) were measured in plasma of pregnant women living in the city of Yaoundé (**A-C**) and village of Ngali II (**D–F**). Samples were randomly selected within a trimester so only one data point per women per trimester was included. Number of samples per trimester ranged from: **A–C**: Primigravidae (PG) n = 33–39, multigravidae (MG) n = 63–74; **D–F**: PG n = 13–15, MG n = 33–68. To determine if Ab levels increased during pregnancy, data for women with samples collected in the first, second, and third trimesters (City: PG n = 32, MG n = 53; Village: PG n = 7, MG n = 17) were assessed using the Friedman's test. As expected, Ab levels to FV2 increased during pregnancy in the village (Fig. **1B**: PG: p = 0.008, MG p = 0.001). Although a similar trend was observed in the city, the increase was not significant, probably due to low transmission (**Fig. 1A**). Likewise, higher Ab levels to FV2 were observed in PG and MG compared to males in the city (all p values <0.001) and village (all p values <0.0001). In contrast, Ab levels to ID1-ID2a (both strains) did not increase during pregnancy in either location (p>0.05) and no significant differences were found in Ab levels to ID1-ID2a (3D7 or FCR3) between males and females in the two sites (all p values>0.05) (Fig. 1B–1F). Although statistically significant higher Ab levels to ID1-ID2a 3D7 were found in MG in the village compared to males (Fig. **E**, p<0.05), the trend was not found with the FCR3 strain. ♂- males, small ♀- PG, big ♀- MG; I, II, III trim  =  first, second and third trimester, respectively. Median MFI and Inter-Quartile Range (IQR) are plotted.

In the initial study, plasma samples collected longitudinally throughout pregnancy from women in whom we had found a correlation between absence of PM and 1) high avidity Ab to FV2 and 2) high Ab levels to multiple VAR2CSA domains [23], [24] were used. We sought to determine if naturally acquired Ab levels to ID1-ID2a correlated with protection from PM. IgG to FV2 (FCR3) and ID1-ID2a (3D7 and FCR3) were measured in women living in Yaoundé (Fig 1. A–C) and Ngali II (Fig. 1 D–F). As expected, Ab levels to FV2 were pregnancy-associated (*i.e.*, Ab levels were significantly higher in pregnant women compared to males living in Yaoundé (all p values <0.001) and Ngali II (all p values <0.0001)). Additionally, IgG to FV2 increased during the course of pregnancy in Ngali II, as women became repeatedly infected with malaria and boosting occurred (primigravidae p = 0.008 and multigravidae p = 0.001, Friedman's test). A similar, but non- significant, trend was also found in women in the city. In contrast, Ab levels to both the 3D7 and FCR3 stains of ID1-ID2a did not change during pregnancy in women living in either Yaoundé or Ngali II (all four p values>0.05). Additionally, Ab levels to ID1-ID2a in pregnant women did not differ significantly from those of sympatric males at both sites (all p values>0.05). Although multigravidae in Ngali II had slightly higher IgG levels to the 3D7 strain of ID1-ID2a than males (p<0.05), the difference was not found with the FCR3 strain of ID1-ID2a. Next, data were stratified by PM status and no significant differences were found in Ab levels to either the 3D7 or FCR3 strain of ID1-ID2a between PM+ and PM- women at delivery, both in the city of Yaoundé (Figure S3A) and Ngali II (Figure S3B) (all p values>0.05). Results indicate that Ab to ID1-ID2a do not increase during pregnancy in women repeatedly exposed to malaria, were not pregnancy-specific, and were not associated with the absence of PM at delivery.

### IgG levels to ID1-ID2a and FV2 in additional subjects

**Figure 2:**
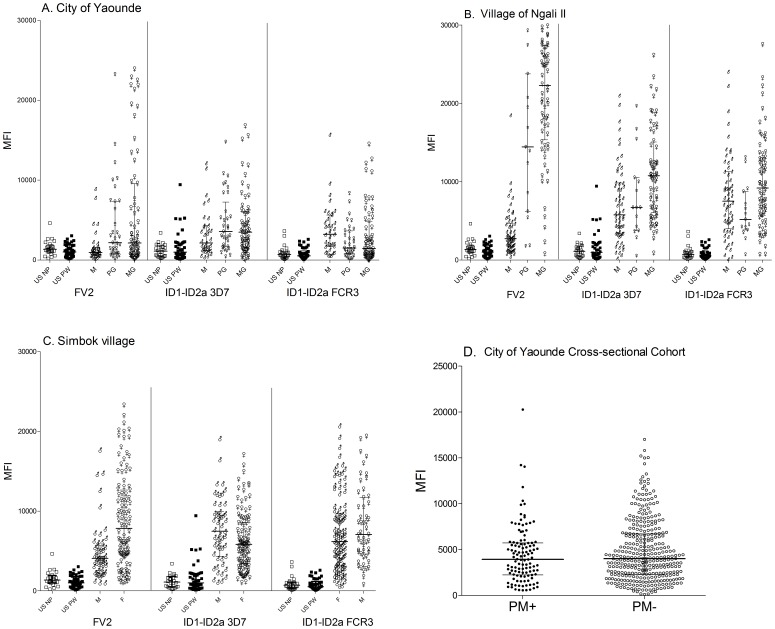
IgG levels to ID1-ID2a in different cohorts. IgG levels to FV2 and ID1-ID2a (3D7 and FCR3) were measured in plasma collected at delivery from women living in **A**) city of Yaoundé (n = 35 males, n = 33 PG, n = 63 MG), **B**) village of Ngali II (n = 58 males, n = 15 PG, n = 68 MG), **C**) Simbok village (n = 51 males, n = 102 women, and **D**) in a cross-sectional cohort of MG in Yaoundé (PM+n = 116, PM− n = 348), as well as, US pregnant (n = 42) and non-pregnant subjects (n = 24). Major findings included: 1) significantly higher Ab levels to ID1-ID2a (both strains) in Cameroonian males (city and village) compared to US pregnant women (p<0.001); 2) similar IgG levels to FV2 in US pregnant and non-pregnant controls and males in Yaoundé; but, higher levels in Cameroonian males living in the village (p<0.0001), suggesting repeated infection induced Ab to FV2 in some males; 3) no significant differences in IgG to ID1-ID2a in Cameroonian males and females living in the city and village (both strains p> values 0.05); and 4) no significant differences in IgG to ID1-ID2a FCR3 between PM+ and PM- MG in the city (**D**: p = 0.46). Horizontal bars represent median, first and third quartiles, and vertical bars represent Inter-Quartile Range (IQR). □- US non-pregnant (US NP), ▪ US pregnant women (US PW), ♂- Cameroonian males (M), small ♀- Cameroonian PG, big ♀- Cameroonian MG. Median MFI and IQR are plotted.

**Figure 3:**
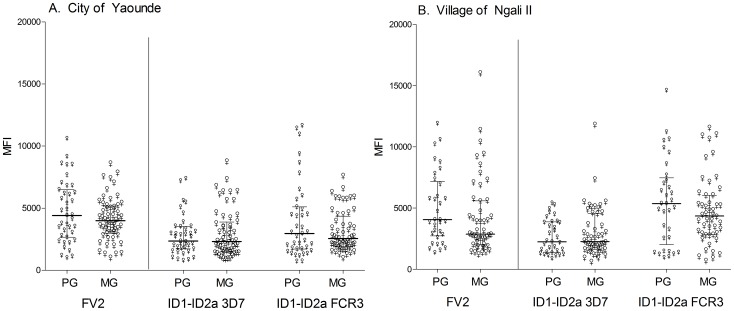
IgM levels to FV2 and ID1-ID2a in Cameroonian women. IgM levels to FV2 and ID1-ID2a (3D7 and FCR3) were measured in plasma collected at first smear-positive visit and the following visit approximately one month later from women living in **A**) city of Yaoundé (PG n = 17, MG n = 26) and **B**) village of Ngali II (PG n = 21, MG n = 33). No statistically significant differences in IgM levels to either ID1-ID2a or FV2 were observed between PG and MG in both locations (all p values>0.05). Since the beads were coupled at saturation, it appears that both PG and MG produced higher IgM levels to FV2 compared to ID1-ID2a 3D7 (both locations p-values <0.001). Yaoundé PG and MG produced higher IgM levels to FV2 compared to ID1-ID2a FCR3 (p<0.05). Small ♀- PG, big ♀- MG. Median MFI and IQR are plotted.

**Figure 4:**
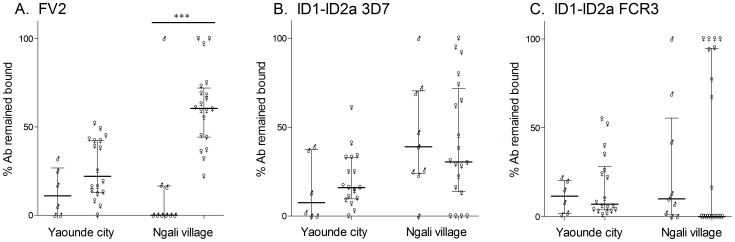
Proportion of high avidity IgG to FV2 and ID1-ID2a. A subset of women in Yaoundé and Ngali II with Ab ID1-ID2a were tested in the avidity assay. The proportion of Ab that remained bound to **A**) FV2, **B**) ID1-ID2a 3D7, and **C**) ID1-ID2a FCR3 in the presence of chaotropic salt was measured in samples collected in city of Yaoundé (n = 6 males, n = 18 women) and village of Ngali II (n = 9 males, n = 20 women). Although the mean proportion of high avidity Ab to FV2 was significantly higher in Ngali II pregnant women compared to males (*** p = 0.0003), no statistically significant differences were found in Ab avidity to ID1-ID2a (3D7 and FCR3) between males and females, both in the city and village (all p>values 0.05). ♂- males, ♀- females Median and IQR are also plotted.

Since the above results were unexpected, additional plasma samples were tested for IgG to ID1-ID2a, including samples from 1) 66 USA subjects including pregnant women, 2) 96 pregnant women in Yaoundé, including primigravide (PG) and multigravidae (MG), collected at delivery and 35 males in Yaoundé (Fig. 2A), and 83 pregnant women and 58 males in Ngali II village (Fig. 2B, Fig. 3) 102 women and 51 males of reproductive age living in Simbok village (Fig. 2C), and (Fig. 4) a cohort of MG (≥G3) in Yaoundé who were PM+ (n = 116) and PM− (n = 348) (Fig. 2D). The results confirmed the initial observation. Major findings included: 1) Ab to ID1-ID2a were absent in US adults and pregnant women and present in equal amounts in Cameroonian males, PG and MG living both in the city of Yaoundé, Ngali II and Simbok (Fig. 2A–C), suggesting that Ab to ID1-ID2a were induced by immunogens or pathogens common in Cameroon that are absent in the USA; 2) similar IgG levels to ID1-ID2a were observed in Cameroonian males and females living in the city and villages (all p values>0.05), showing that the response was ubiquitous; and 3) similar IgG levels to ID1-ID2a were found in PM+ and PM− MG from the large cohort of women living in Yaoundé (p = 0.46) (Fig. 2D). Again, the only suggestion that Ab to ID1-ID2a might be induced during pregnancy was that MG, but not PG, in Ngali II had higher Ab levels to the 3D7, but not FCR3, strain of ID1-ID2a compared to males (p<0.0001).

For comparison, the above samples were screened against FV2 (Fig. 2A–C). As previously reported [23], Ab levels to FV2 were significantly higher in PG and MG women compared to males both in the city of Yaoundé and Ngali II (all p values <0.05) (Fig. 2A–C). Thus, the Ab response to FV2 was pregnancy-associated; whereas, the response to ID1-ID2a was not.

The prevalence of Ab to ID1-ID2a is shown in Table 1. Although Ab to FV2 were essentially absent in males and present in a high proportion of PG (Yaoundé: 39%, Ngali II: 73%) and MG (Yaoundé: 38%, Ngali II: 93%); the prevalence of Ab to ID1-ID2a did not differ significantly among Cameroonian males, PG and MG (3D7 Yaoundé: p = 0.1; FCR3 Yaoundé: p = 0.21, FCR3 Ngali II: p = 0.21), except for the 3D7 strain in Ngali II (p = 0.0013). Overall, 60% of males in the city of Yaoundé, 90% in Ngali II, and 96% in Simbok village had Ab to at least one of the two ID1-ID2a variants; while, <11% of the males in Yaoundé and the rural villages were seropositive for FV2 (Table 1). Thus, most Cameroonian adults have Ab to ID1-ID2a.

**Table 1:** Prevalence of IgG to FV2 and ID1-ID2a in non-pregnant individuals and pregnant women at delivery.

			Antibody Prevalence (%)			
	N	Age^d^	FV2^a^	ID1-ID2a 3D7^b^	ID1-ID2a FCR3^b^	ID1-ID2a FCR3 or 3D7
US non-pregnant	24	-	0	0	8.0	8.0
US pregnant	42	28±7	0	9.5	2.4	9.5
**Yaoundé (city) - longitudinal**						
Males	33	-	11	31	60	60
Primigravidae	33	18±2	39	49	42	49
Multigravidae	63	27±7	38	43	44	44
**Yaoundé (city) - cross-sectional**						
All Multigravidae	464		95	-	63	63
PM+ Multigravidae	116	28±5	86	-	60	60
PM- Multigravidae	348	30±6	97^c^	-	63	63
**Ngali II (village) - longitudinal**						
Males	58	-	8.6	69	88	90
Primigravidae	15	21±4	73	73	93	93
Multigravidae	68	27±5	93	94	96	96
**Simbok (village) – cross-sectional**						
Males	51	26±5	7.8	80	96	96
Females	102	26±5	34	71	87	91
FV2-seropositive^c^	35	27±5	100	97	97	97
FV2-seronegative	50	26±5	0	58	82	88

a Cut-off for FV2 seropositivity was determined based on the mean + 2 SD for resident males after excluding the occasional male (n = 2 Yaoundé, n = 1 Ngali, n = 1 Simbok) with MFI greater than the mean +3 SD.

b US controls (n = 66) were used to establish a cut-off for seropositivity to ID1-ID2a.

c 3% of women who were not seropositive for FV2 were seropositive for DBL5 FCR3, indicating they had been exposed to CSA-binding *P. falciparum* IE.

d Mean ± SD.

Since, the majority of the IgG Ab response to ID1-ID2a was not pregnancy-associated, but Ab levels to the 3D7 strain of ID1-ID2a were slightly higher in MG compared to males in Ngali II, we sought to determine if: 1) ID1-ID2a induced a pregnancy-associated IgM response without isotype-switching to IgG, i.e., was a weak immunogen, 2) Ab avidity to ID1-ID2a differed between MG and males, and 3) MG have Ab to epitopes in other N-terminal DBL domain constructs that are not present in males.

### IgM levels to FV2 and ID1-ID2a

IgM to ID1-ID2a was measured since it is possible epitopes in the ID1-ID2a region of VAR2CSA are poorly immunogenic and/or malaria parasites use “IgM masking” [31] to evade immune response to the functionally important part of the VAR2CSA. Since IgM Ab quickly wane, IgM Ab to ID1-ID2a were measured in plasma of PG and MG living in city of Yaoundé and Ngali II village (longitudinal study) when they were blood-smear positive and one month later. No statistically significant differences were observed between PG and MG IgM levels to any of the antigens in the city (Fig. 3A) and village (Fig. 3B), all p values >0.05. Since beads used in the assay were run at saturation, it appears women in the city produced significantly higher IgM levels to FV2 than ID1-ID2a (both 3D7and FCR3) (Fig. 3A: all p values <0.05) when infected. In Ngali II, both PG and MG produced significantly higher IgM to FV2 than ID1-ID2a 3D7, but not ID1-ID2a FCR3. Although the IgM response to ID1-ID2a was low compared to FV2, there was no evidence that women produced strong IgM, without IgG responses, to ID1-ID2a. Thus, the absence of a pregnancy-associated response to ID1-ID2a was not due to failure in isotype- switching.

### Antibody avidity of Ab to ID1-ID2a

Since IgG Ab to ID1-ID2a appeared to be due to cross-reactive epitopes, we speculated that Ab in males to ID1-ID2a might be low avidity; whereas, a proportion of the Ab in pregnant women would be high avidity if they were induced by VAR2CSA. A subset of longitudinally-collected plasma samples from Yaoundé and Ngali II with Ab to ID1-ID2a were used to measure the proportion of high avidity Ab to ID1-ID2a. The proportion of high avidity Ab to ID1-ID2a 3D7 and FCR3 (Fig. 4B and 4C, respectively) did not differ between males and females living in Yaoundé or Ngali II (all p values>0.05). In contrast, women in Ngali II have a higher proportion of high avidity Ab to FV2 compared to Ngali II males (p = 0.0003), and a similar, but not statistically significant trend was observed in Yaoundé. Finally, we determined the proportion of individuals with high avidity Ab to ID1-ID2a (i.e., ≥35% Ab that remained bound in the presents of a chaotropic salt). In the village, a significantly higher proportion of women had high avidity Ab to FV2 compared to males (90% vs. 11%, p<0.0001) with a similar trend in the city (44% vs. 0%, p = 0.066). However, the proportion of men and women with high avidity Ab to both recombinant ID1-ID2a domains was similar, both in the city and the village (3D7: 56% vs 45%, FCR3: 33% vs. 40%). Interestingly, in the Ngali II about 56% of males had high avidity Ab to ID1-ID2a; whereas only11% had high avidity Ab to FV2. These results suggest affinity maturation occurs in MG in response to FV2; whereas, high affinity Ab to ID1-ID2a were often acquired by Cameroonian adults, but the response lacks the strong parity-related seroepidemiology seen with Ab to FV2.

### Comparison of Ab levels to ID1-ID2a and FV2

**Figure 5:**
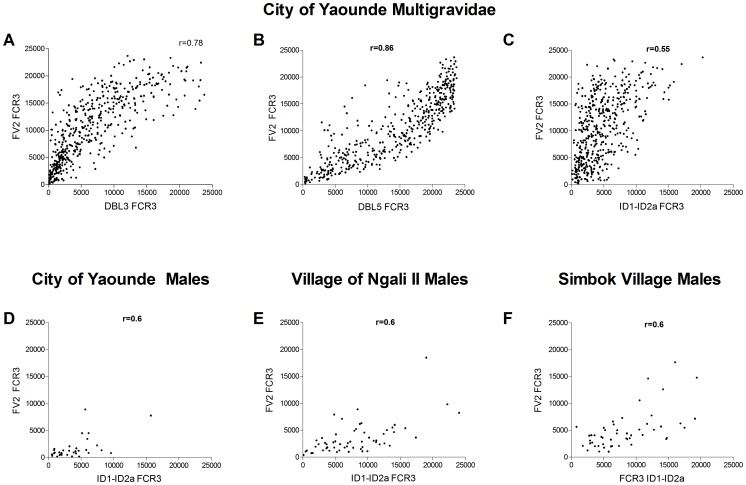
Comparison of Ab levels between FV2 and ID1-ID2a. **A–C**) Ab levels to FV2, DBL3, DBL5 and ID1-ID2a FCR3 were measured in plasma of 116 PM+ and 348 PM- pregnant women living in city of Yaoundé. In addition, comparisons were made in Ab levels to FV2 and ID1-ID2a in **D**) males in Yaoundé, **E**) males in the village of Ngali II, and **F**) males in Simbok village. Positive correlations were found for all comparisons; Pearson's correlation coefficients (r) are reported.

Since Ab to FV2 are induced only in pregnant women, the correlation between the IgG levels to ID1-ID2a and FV2 was analyzed. For comparison, the FCR3 stain of DBL3 and DBL5, that are known to be highly immunogenic and pregnancy-associated, were included in the analysis. Data for Ab to DBL3 and DBL5 (Fig. 5A and 5B) are highly correlated with Ab to FV2 (DBL3 r = 0.78, DBL 5 r = 0.86); however, only a moderate positive correlation was found between Ab to FV2 and ID1-ID2a (FCR3) (r = 0.55) (Fig. 5C) with similar correlations observed in males and females in the city and village (all r = 0.60) (Fig. 5 C–E). Thus, Ab to ID1-ID2a in both males and females have a modest correlation with Ab to FV2.

### IgG levels to the N-terminal domains and DBL5

**Figure 6:**
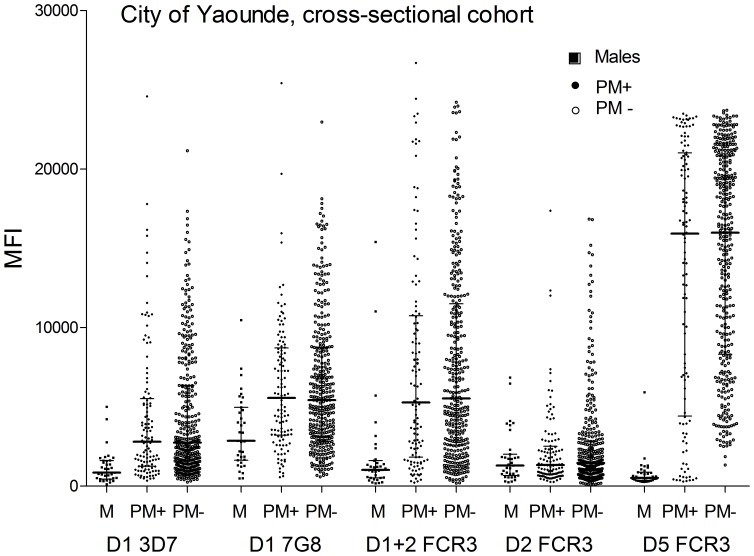
IgG levels to N-terminal domains of FV2 and DBL5 in the city of Yaoundé. Ab levels to DBL1 (3D7 and 7G8), DBL1+2 (7G8), DBL2 FCR3 and DBL5 FCR3 in the plasma of 116 PM+ and 348 PM− multigravidae women living in the city of Yaoundé were measured at delivery (cross-sectional cohort). Both PM+ and PM− women had significantly higher levels of Ab to DBL5 compared to Ab to DBL1, DBL 1+2 and DBL 2 (all p values <0.0001). PM+ and PM− multigravidae had significantly higher levels of Ab to DBL1 3D7, DBL1 7G8, DBL1+2 and DBL5 compared to males (all p values <0.0001). Only a marginally significant differences was found in Ab levels to DBL2 between males and females (p = 0.051). No statistical differences were found in Ab levels to any of the domains between PM+ and PM− women (all p values>0.05). Open circles represent PM− and black circles represent PM+. Median and IQR are also plotted.

Since Ab levels to ID1-ID2a were low compared to FV2, DBL3 and DBL5, the IgG response to other N-terminal domains that contain portions of the ID1-ID2a region were assessed using samples from the Yaoundé cross-sectional cohort (Fig. 6) and from individuals living in Ngali II (Fig. S4). IgG levels to DBL1 (3D7), DBL1 (7G8), DBL2 1+2 (7G8), DBL2 (FCR3) and DBL5 (FCR3) were measured in 116 PM+ and 348 PM- MG living in the city of Yaoundé. The Ab response to the N-terminal domains was relatively low compared to DBL5 (*e.g.*, means MFI ranging from 1,335 to 5,566 vs. 15,938 to 15,996). The Ab response of males to DBL1 (3D7 and 7G8 p<0.0001) and DBL1+2 (p<0.0001) was lower than females; whereas, the difference between males and females to DBL2 was only marginally significant (p = 0.051) (Fig. 6). A similar pattern was found using plasma samples from males and females residing in Ngali II cohort (Fig. S4). Thus, Ab levels to the N-terminal region tended to be low and the difference between pregnant women and males was less apparent than for DBL5.

## Discussion

This study evaluated the naturally acquired Ab response of pregnant Cameroonian women to ID1-ID2a, a small protein containing the minimal CSA-binding site. Results showed Ab levels to ID1-ID2a did not change during pregnancy, did not increase with gravidity, and were present at similar levels and prevalence in Cameroonian males, primigravidae and multigravidae. IgG Ab levels to ID1-ID2a were generally low compared to FV2; however, there was no evidence this was due to failure in isotype-switching from IgM to IgG. No differences in either Ab avidity to ID1-ID2a or proportion of high avidity Ab was found between Cameroonian males and pregnant women. Our data show that naturally-acquired Ab to ID1-ID2a are not pregnancy-specific or associated with absence of PM, but are cross-reactive and detect epitopes that are normally hidden in intact FV2.

These results were surprising, as we initially hypothesized that naturally acquired Ab levels to ID1-ID2a would be low, but highly pregnancy-specific and correlated with absence of PM, i.e., similar to Ab response to FV2 [23]. Thus, when results from the first longitudinal study suggested our hypothesis was wrong, we studied the Ab responses to ID1-ID2a in larger cohorts from different malaria transmission sites. Data showed that Ab to ID1-ID2a are not present in US adults including pregnant women; however, Ab are present at similar levels in Cameroonian primigravidae, multigravidae and males living both low (Yaoundé) and high (Simbok and Ngali II) malaria transmission settings.

No strong evidence was found that naturally-acquired Ab to ID1ID2a were induced by VAR2CSA. For example, ≤11% of Cameroonian males had Ab to FV2, indicating that few males are exposed to immunogenic levels of VAR2CSA; whereas, 60% of males in Yaoundé, 90% in Ngali II, and 96% in Simbok had Ab to ID1-ID2a (Table 1). Likewise, many primigravidae had Ab to ID1-ID2a during the first trimester prior to producing Ab to FV2 (Fig. 1), and 88% of women in Simbok who were sero-negative for FV2 had Ab to ID1-ID2a (Table 1). The antigen(s) inducing the cross-reactive Ab is unknown. Since adults in the USA lack Ab to ID1-ID2a, the Ab appear to be elicited by endemic pathogens found in Africa and not by pathogens found world-wide or in childhood vaccines (e.g., measles, rubella, diphtheria, poliovirus). The cross-reactive Ab could have been induced by other PfEMP1 molecules or other malarial antigens. Alternatively, *P. falciparum* parasites switch on *var2csa* gene expression and these IE are rapidly cleared in non-pregnant individuals due to lack of cytoadherence. In this case, individuals might be exposed to denatured forms of VAR2CSA during the parasite-clearance process. Since the source of the cross-reactive Ab remains unknown, the presence of cross-reactive Ab needs to be taken into consideration in initial vaccine trials in endemic areas, since cross-reactive Ab might reduce the response to ID1-ID2a by neutralizing ID1-ID2a before it stimulates a humoral response or enhance the response to ID1-ID2a by stimulating pre-existing T helper cells. In addition, adverse effects could potentially arise due to boosting of cross-reactive responses that cause immune-complex mediated pathology.

The data provide little evidence that Cameroonian women produce Ab to epitopes within the minimal CSA-binding site as a result of natural infection. This result contrasts with the strong Ab response elicited in rats. Immunization studies, conducted using ID1-ID2a in malaria-naïve rats, resulted in strong inhibition of binding activity of IE to CSA [22]; whereas, humans develop Ab to cross-reactive B cell epitopes normally hidden in full-length VAR2CSA. Since rats lacked Ab to these epitopes, their immune response could be directed toward the binding site. It is interesting that other constructs containing the CSA-binding sites, i.e., DBL1 and DBL2, were also poorly immunogenic in humans (Fig. 6). It is possible that pregnant women produce a strong Ab response to DBL3, DBL4, DBL5, and this dominant response diverts the immune response away from the important epitopes in the binding site, providing an immune evasion mechanism. VAR2CSA has six major domains, but the minimal binding site is contained within only one of them. From the evolutionary stand point, parasites could use the other domains to direct the immune response to other parts of the FV2, and as a result, a very non-immunogenic region in DBL2 has evolved. Only parasites with non-immunogenic DBL2 would have a selective advantage for survival in semi-immune hosts. Clearly, additional studies are needed to accept or refute this theory.

Although the majority of the data showed no statistically-significant differences between Ab responses to ID1-ID2a in pregnant women and males, there was one exception, namely, a statistically-significant increase in Ab levels to the 3D7 strain of ID2-ID2a in multigravidae compared to males in the village of Ngali II. This difference was not found in pregnant women the city or in non-pregnant women in a cross-sectional study in Simbok, which is also a high transmission site. Based on these results, one cannot rule out the possibility that after multiple infections pregnant women produce Ab to strain-specific epitopes in ID1-ID2a that persist for a short period of time. If true, a vaccine to ID1-ID2a might be able to induce higher Ab levels that would persist longer and provide protection from PM. Unfortunately, there was no indication that Ab to ID1-ID2a were associated with absence of PM at delivery.

Clearly, significant progress has been made toward the development of a vaccine that would protect an estimated 85 million women world-wide who are exposed to *P. falciparum* during pregnancy [32]. The discovery that IE bind to CSA in the placenta provided a key explanation as to why *P. falciparum*-infected erythrocytes accumulate at the feto-maternal interface, causing PM [12]. The subsequent identification of VAR2CSA as the parasite antigen responsible for the binding was a critical step toward the development of a vaccine for prevention of PM [3]. Since VAR2CSA is a large molecule, studies identifying the minimal sequence that binds to CSA were highly important [20], [21], [33]. The study reported herein is the first to evaluate the Ab response of pregnant women living in a malaria-endemic to the recombinant ID1-ID2a vaccine candidate that is currently undergoing clinical Phase I evaluation. Our data indicate that acquisition of Ab to the ID1-ID2a protein after natural infection is not parity-dependent, but rather a “high” background level of cross-reactive Ab exists in the general population. Although, these data suggest that naturally acquired protection from PM is not mediated by ID1-ID2a, data from animal studies demonstrate that the vaccination-induced Ab to recombinant ID1-ID2a may be protective. Currently, the ID1-ID2a development group is trying to develop a vaccine that elicits high levels of protective Ab to recombinant ID1-ID2a by making the vaccine more immunogenic. Clinical trials are necessary to determine the efficacy of ID1-ID2a as a recombinant subunit vaccine.

Testing first-generation VAR2CSA-based vaccines in pregnant women is going to be a challenge. It is therefore important to understand the characteristics of Ab that mediate protection and have serological assays that accurately measure them. Although Ab most likely mediate their effect by preventing the binding of IE to CSA, this has been difficult to firmly establish in women with naturally acquired immunity. The data reported herein demonstrated that Ab levels to these recombinant ID1-ID2a proteins cannot be used as correlates of protection. The best predictors of protection identified to date are high avidity Ab to FV2 and having Ab to multiple DBL domains [23], [24]; however, better predictors are needed. Additional approaches should be explored for determining the immune status of pregnant women for PM, for example, statistical models using a combination of serological assays or evaluating functional assays, such as phagocytosis and inhibition of binding, should be investigated.

## Supporting Information

Figure S1 **Sequence of ID1-ID2a recombinant protein.** Sequence of ID1-ID2a protein from 3D7 strain (A) and FCR3 strain (B) used in the study. Sequence boundaries for 3D7 ID1-ID2a are N385 to D1017 and for FCR3 ID1-ID2a are N386 to D1025.
(DOCX)

Figure S2 **ID1-ID2a and FV2 used alone or combined in the MAP assay.** Microspheres coupled with ID1-ID2a (3D7 and FCR3) were tested alone and combined with FV2 FCR3 in the MAP assay using a pool of plasma from pregnant women with high Ab titers to VAR2CSA. Mean MFI ± SD are shown. No significant differences were observed between Ab levels when the antigens were used alone or multiplexed, except a minor increase in Ab levels to FV2 FCR3 when it was multiplexed with ID1-ID2a 3D7 (p = 0.032).
(TIF)

Figure S3 **IgG levels to ID1-ID2a in Cameroonian PM+ and PM- women at delivery.** IgG levels to both strains of ID1-ID2a were measured in plasma collected at delivery from women living in A) the city of Yaoundé (PG n = 12PM−, n = 8PM+; MG n =  33PM−, n = 11PM+) and B) Ngali II village (PG n = 3PM−, n = 7PM+; MG: n = 28 PM−, n = 32 PM+) for whom PM status was known. The antibody levels to the 3D7 and FCR3 strains of ID1-ID2a were not statistically different between PM+ and PM- women (Yaoundé: 3D7 PG p = 0.2, MG p = 0.19; FCR3 PG p = 0.027, MG p = 0.38; Ngali 3D7 PG p = 1, MG p = 0.45; FCR3 PG p = 0.83. MG p = 0.39). Horizontal bars represent median and whiskers represent IQR.
(TIF)

Figure S4 **IgG levels to N-terminal domains of FV2 and DBL5 in the village of Ngali II.** IgG levels to the N-terminal domains, i.e., DBL1 (3D7 and 7G8), DBL1+2 (7G8), DBL2 FCR3 as well as DBL5 FCR3 were measured in plasma collected at delivery from women residing in Ngali II. The current analysis includes only those women for who PM status was known (PG n = 3 PM−, n = 7 PM+; MG n = 28 PM−, n = 32 PM+) and 20 Ngali males. No significant differences were found between PM+ and PM- primigravidae (D1 3D7 p = 0.83, D1 7G8 p = 1, D1+2 p = 1, D2 p = 1, D5 p = 0.67) or multigravidae (D1 3D7 p = 0.12, D1 7G8 p = 0.07, D1+2 p = 32, D2 p = 0.11, D5 p = 0.44). Statistically significant differences between Ngali PM+ and PM− PG and males were observed for only DBL1+2 (PM+ vs. males p = 0.03) and DBL5 (PM+ p = 0.0003, PM− p = 0.04). PM− and PM+ MG had significantly higher levels of Ab to DBL1+2, DBL2 and DBL5 compared to males (PM+ p<0.0001, p = 0.0003, p<0.0001 and PM− p<0.0001, p = 0.02, p<0.0001 respectively). Horizontal bars represent median and whiskers represent IQR.
(TIF)
