# Supplementary material for: The Antibody Response of Pregnant Cameroonian Women to VAR2CSA ID1-ID2a, a Small Recombinant Protein Containing the CSA-Binding Site
Source: PLoS One. 2014 Feb 4;9(2):e88173. doi: 10.1371/journal.pone.0088173 (PMC3913775; doi:10.1371/journal.pone.0088173)
Supplement: Figure S1 — Sequence of ID1-ID2a recombinant protein. Sequence of ID1-ID2a protein from 3D7 strain (A) and FCR3 strain (B) used in the study. Sequence boundaries for 3D7 ID1-ID2a are N385 to D1017 and for FCR3 ID1-ID2a are N386 to D1025. (DOCX) [file pone.0088173.s001.docx]

**Figure S1.**

1. **Sequence of ID1-ID2a, 3D7 strain**

**Sequence Boundaries:** N385 to D1017

NYIKGDPYFAEYATKLSFILNSADANNPSEKIQKNNDEVCNCNESGIASVEQEQISDPSSNKACITHSSIKANKKKVCKHVKLGVRENDKDLRVCVIEHTSLSGVENCCCQDFLRILQENCADNKAGSSSNGACNNKNQEACEKNLEKVLASLTNCYKCDKCKSEQSKKNNKNWIWKKSSGKEGGLQKEYANTIGLPPRTQSLCLVVCLDEKGKKTQELKNIRTNSELLKEWIIAAFHEGKNLKPSHEKKNDDNGKKLCKALEYSFADYGDLIKGTSIWDNEYTKDLELNLQKIFGKLFRKYIKKNNTAEQDTSYSSLDELRESWWNTNKKYIWLAMKHGAGMNSTTCCGDGSVTGSGSSCDDIPTIDLIPQYLRFLQEWVEHFCKQRQEKVKPVIENCKSCKESGGTCNGECKTECKNKCEVYKKFIEDCKGGDGTAGSSWVKRWDQIYKRYSKYIEDAKRNRKAGTKNCGPSSTTNAAENKCVQSDIDSFFKHLIDIGLTTPSSYLSIVLDDNICGADKAPWTTYTTYTTTEKCNKETDKSKLQQCNTAVVVNVPSPLGNTPHGYKYACQCKIPTNEETCDDRKEYMNQWSCGSARTMKRGYKNDNYELCKYNGVDVKPTTVRSNSSKLD

1. **Sequence of ID1-ID2a,** **FCR3 strain**

**Sequence Boundaries:** N386 to D1025

NYIKGDPYFAEYATKLSFILNPSDANNPSGETANHNDEACNCNESGISSVGQAQTSGPSSNKTCITHSSIKTNAAAECADVKLGVRENDKDLKICVIEDTSLSGVDNCCCQDLLGILQENCSDNKRGSSSNDSCDNKNQDECQKKLEKVFASLTNGYKCDKCKSGTSRSKKKWIWKKSSGNEEGLQEEYANTIGLPPRTQSLYLGNLPDINFDTKEKFLAGCLIVSFHEGANLAAAYPQNKNSGNKENLCKALEYSFADYGDLIKGTSIWDNEYTKDLELNLQNNFGKLFGKYIKKNNTAEQDTSYSSLDELRESWWNTNKKYIWTAMKHGAEMNITTCNADGSVTGSGSSCDDIPTIDLIPQYLRFLQEWVENFCEQRQAKVKDVITNCKSCKESGNKCKTECKTKCKDECEKYKKFIEACGTAGGGIGTAGSPWSKRWDQIYKRYSKHIEDAKRNRKAGTKNCGTSSTTNAAASTDENKCVQSDIDSFFKHLIDIGLTTPSSYLSNVLDDNICGADKAPWTTYTTYTTTEKCNKERDKSKSQSSDTLVVVNVPSPLGNTPYRYKYACQCKIPTNEETCDDRKEYMNQWSCGSARTMKRGYKNDNYELCKYNGVDVKPTTVRSNSSKLD
