# Supplementary figures and images for: The Antibody Response of Pregnant Cameroonian Women to VAR2CSA ID1-ID2a, a Small Recombinant Protein Containing the CSA-Binding Site
Source: PLoS One. 2014 Feb 4;9(2):e88173. doi: 10.1371/journal.pone.0088173 (PMC3913775; doi:10.1371/journal.pone.0088173)

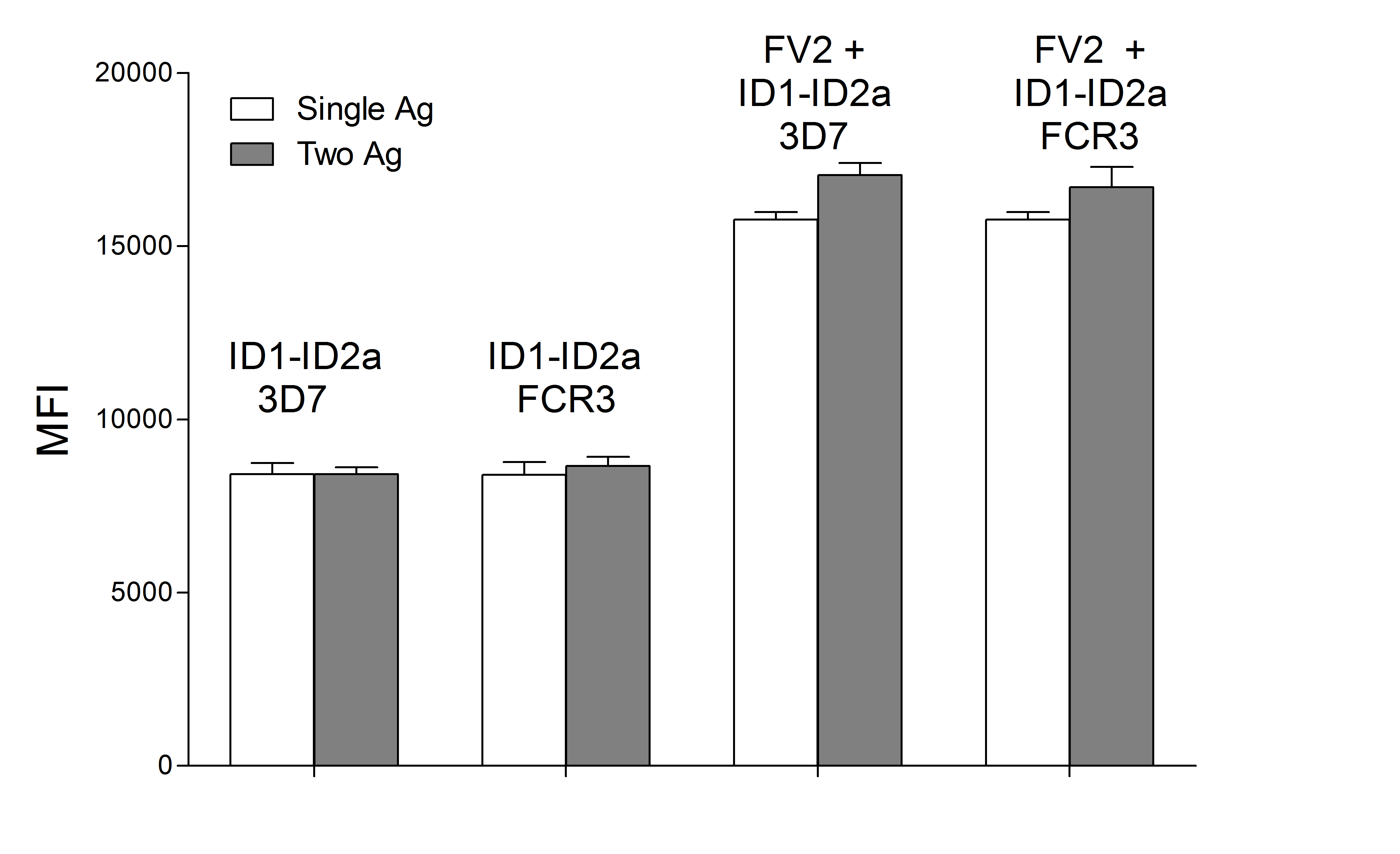

Supplement: Figure S2 — ID1-ID2a and FV2 used alone or combined in the MAP assay. Microspheres coupled with ID1-ID2a (3D7 and FCR3) were tested alone and combined with FV2 FCR3 in the MAP assay using a pool of plasma from pregnant women with high Ab titers to VAR2CSA. Mean MFI ± SD are shown. No significant differences were observed between Ab levels when the antigens were used alone or multiplexed, except a minor increase in Ab levels to FV2 FCR3 when it was multiplexed with ID1-ID2a 3D7 (p = 0.032). (TIF) [file pone.0088173.s002.tif]

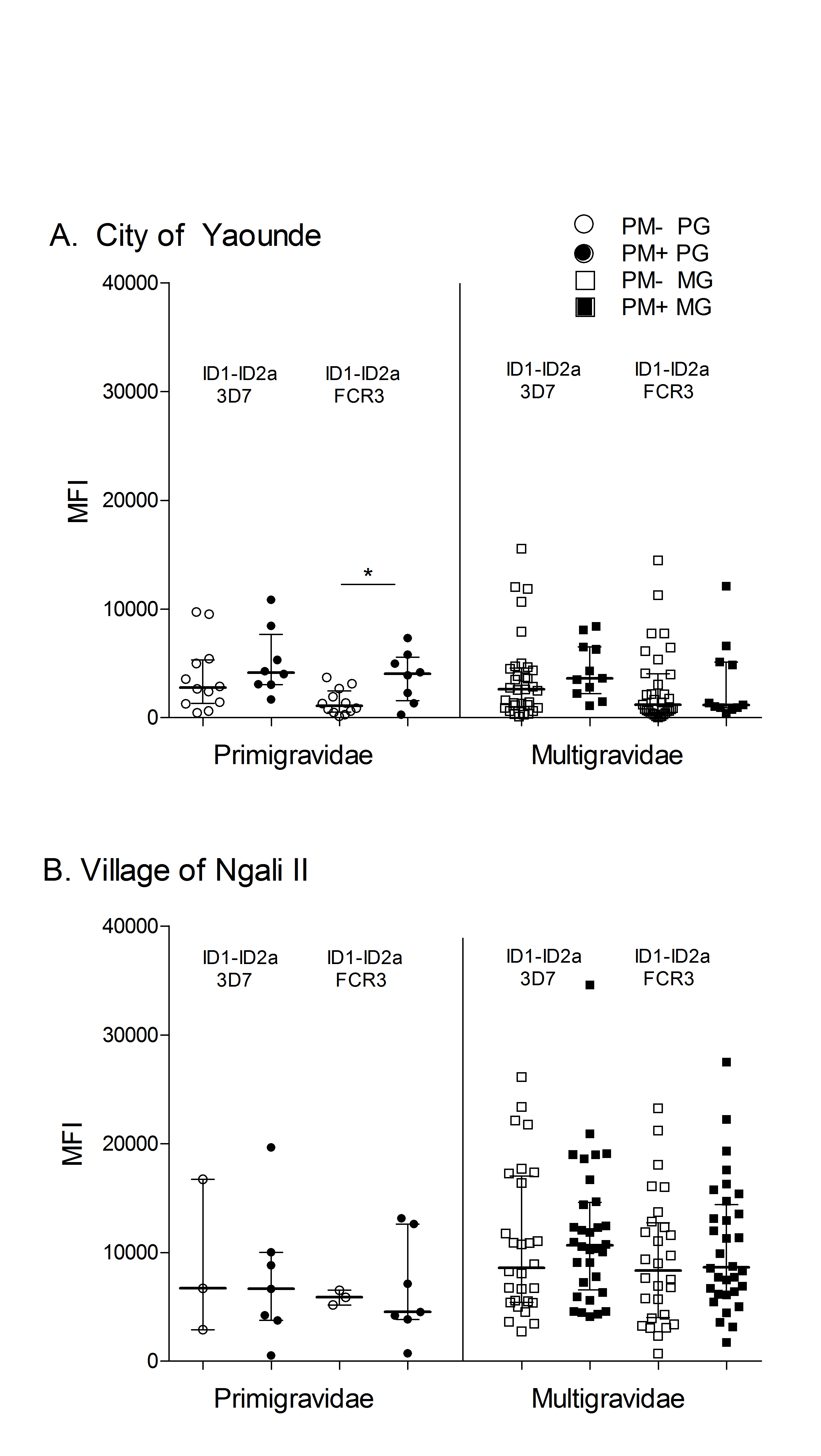

Supplement: Figure S3 — IgG levels to ID1-ID2a in Cameroonian PM+ and PM- women at delivery. IgG levels to both strains of ID1-ID2a were measured in plasma collected at delivery from women living in A) the city of Yaoundé (PG n = 12PM−, n = 8PM+; MG n = 33PM−, n = 11PM+) and B) Ngali II village (PG n = 3PM−, n = 7PM+; MG: n = 28 PM−, n = 32 PM+) for whom PM status was known. The antibody levels to the 3D7 and FCR3 strains of ID1-ID2a were not statistically different between PM+ and PM- women (Yaoundé: 3D7 PG p = 0.2, MG p = 0.19; FCR3 PG p = 0.027, MG p = 0.38; Ngali 3D7 PG p = 1, MG p = 0.45; FCR3 PG p = 0.83. MG p = 0.39). Horizontal bars represent median and whiskers represent IQR. (TIF) [file pone.0088173.s003.tif]

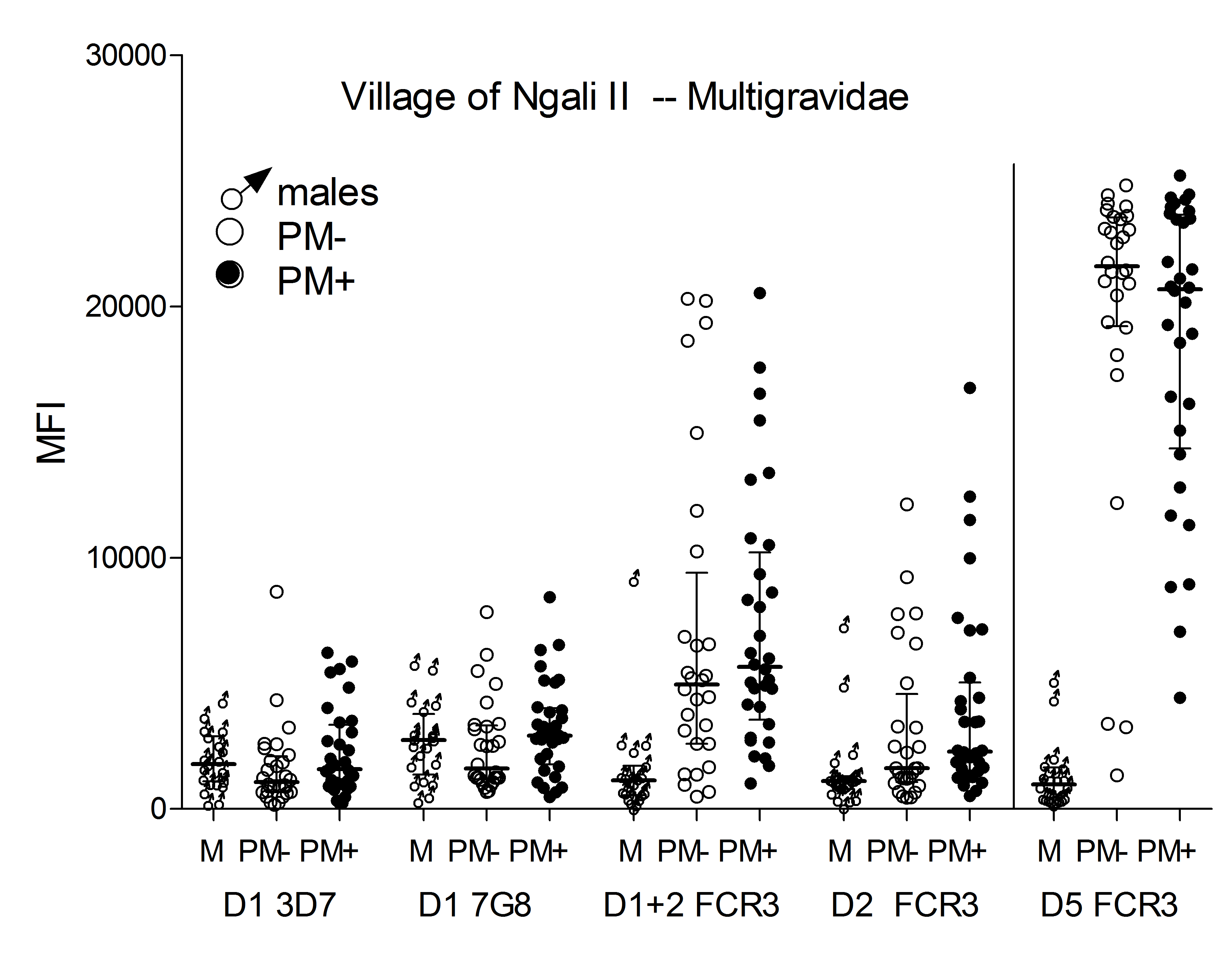

Supplement: Figure S4 — IgG levels to N-terminal domains of FV2 and DBL5 in the village of Ngali II. IgG levels to the N-terminal domains, i.e., DBL1 (3D7 and 7G8), DBL1+2 (7G8), DBL2 FCR3 as well as DBL5 FCR3 were measured in plasma collected at delivery from women residing in Ngali II. The current analysis includes only those women for who PM status was known (PG n = 3 PM−, n = 7 PM+; MG n = 28 PM−, n = 32 PM+) and 20 Ngali males. No significant differences were found between PM+ and PM- primigravidae (D1 3D7 p = 0.83, D1 7G8 p = 1, D1+2 p = 1, D2 p = 1, D5 p = 0.67) or multigravidae (D1 3D7 p = 0.12, D1 7G8 p = 0.07, D1+2 p = 32, D2 p = 0.11, D5 p = 0.44). Statistically significant differences between Ngali PM+ and PM− PG and males were observed for only DBL1+2 (PM+ vs. males p = 0.03) and DBL5 (PM+ p = 0.0003, PM− p = 0.04). PM− and PM+ MG had significantly higher levels of Ab to DBL1+2, DBL2 and DBL5 compared to males (PM+ p<0.0001, p = 0.0003, p<0.0001 and PM− p<0.0001, p = 0.02, p<0.0001 respectively). Horizontal bars represent median and whiskers represent IQR. (TIF) [file pone.0088173.s004.tif]
